# Structure of Waste Hemp Stalks and Their Sound Absorbing Properties

**DOI:** 10.3390/polym14224844

**Published:** 2022-11-10

**Authors:** Shuang Su, Yuan Gao, Xinghai Zhou, Xiaoqing Xiong, Ying Wang, Lihua Lyu

**Affiliations:** School of Textile and Material Engineering, Dalian Polytechnic University, Dalian 116034, China

**Keywords:** waste hemp stalk, structural characteristics, sound-absorbing properties, sound-absorbing mechanism, composite materials

## Abstract

To broaden the application fields of waste hemp stalks, the macromolecular, supramolecular, and morphological structures of waste hemp stalks were analyzed, and the relationship between these properties and the sound absorption properties of the hemp stalks was explored. Then, waste hemp stalk/polycaprolactone sound-absorbing composite materials were prepared by the hot pressing method. The influence of hemp stalk length and mass fraction, and the density and thickness of the composite materials on the sound absorption properties of composites prepared with the hot pressing temperature set to 140 °C, the pressure set to 8 MPa, and the pressing time set to 30 min was investigated. The results showed that, when the sound energy acts on the hemp stalk, the force between the chain segments, the unique hollow structure, and the large specific surface, act together to attenuate the sound energy and convert it into heat and mechanical energy in the process of propagation, to produce a good sound absorption effect. When the hemp stalk length and mass fraction were set to 6 mm and 50%, respectively, and the density and thickness of the material were set to 0.30 g/cm^3^ and 1.5 cm, respectively, the average sound absorption coefficient of the waste hemp stalk/polycaprolactone sound-absorbing composite material was 0.44, the noise reduction coefficient was 0.42, the maximum sound absorption coefficient was 1.00, and the sound-absorbing band was wide. The study provided an experimental and theoretical basis for the development of waste hemp stalk/polycaprolactone sound-absorbing composite materials, and provided a new idea for the recycling of the waste hemp stalk.

## 1. Introduction

Noise pollution is becoming more and more serious, and according to statistics, 42.1% of complaints about the urban environment were related to noise [1]. At the same time, hemp production in 2020 was more than 100,000 tons and the hemp stalks were discarded or burned. In addition to wasting an excellent natural resource, burning waste hemp stalks produces a large amount of carbon dioxide, nitrogen oxides, hydrogen sulfide, soot, etc. [2]. Waste hemp stalks are widely available, low cost, and have a natural hollow structure, and, thus, are suitable for use in sound-absorbing composite materials. Using waste hemp stalks to prepare a sound-absorbing composite material with light weight and high porosity to effectively alleviate the noise pollution problem, not only improves the comprehensive and efficient utilization of biological materials such as hemp stalks but also avoids the disadvantage of environmental pollution caused by burning hemp stalk while expanding the applications of hemp stalks. 

**Figure 1:**
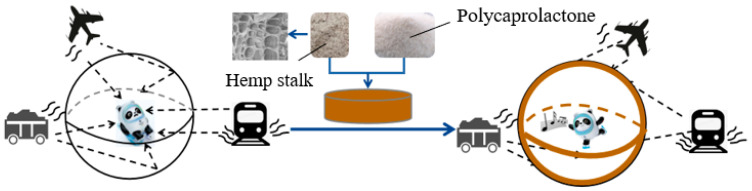
Schematic diagram of research significance and application.

To use renewable biomass resources effectively, the environmentally friendly, high-value utilization of hemp stalks in sound-absorbing material is being investigated for use in effectively alleviating noise pollution. This is of great significance in social development and environmental protection, in line with the current dual “carbon neutral” and “carbon peak” goals that form part of the policy of sustainable development requirements in China and other nations. The schematic diagram of research significance and application is shown in Figure 1.

Xu et al. [3] made needle felt by mixing PLA (poly lactic acid) fiber and hemp fiber at a certain weight ratio and formed hemp/PLA composites through hot pressing. Viel et al. [4] selected three types of hemp stalks to prepare composite materials and analyzed them by chemical analysis and scanning electron microscopy (SEM), and the results showed good thermal properties, which verified the feasibility of preparing composite materials with hemp stalks. Lyu et al. [5] used waste corn husk fibers as reinforcement material and PLA particles as the matrix material to prepare sound-absorbing composite materials and investigated the sound-absorbing properties of multilayer structural composite materials. Samaei et al. [6] studied an innovative natural fiber-granular composite made of kenaf fibers and waste rice husk granules. The results showed that, at all frequencies, the optimized fiber-granular sample provided higher sound absorption performance compared with samples made of 100% kenaf or 100% rice husk. Wang et al. [7] used hemp to prepare composite materials of different thicknesses and diameters and then conducted experiments by the standing wave tube method. They also obtained the sound absorption coefficient values by numerical simulations. The results showed that the composite materials had sound-absorbing properties, and the experiment verified that hemp could be used to prepare sound-absorbing composite materials. Agirgan et al. [8] used stalk fibers to produce new single-layer and bi-layer biocomposite materials by a hot pressing process. The obtained composite materials were characterized using instrumental methods such as SEM. The results showed that the bi-layer composite materials had the highest sound absorption coefficient values, close to 0.99, while the single-layer composite materials had an absorption coefficient of approximately 0.34. This indicates that the thickness of the composite material affects its sound-absorbing properties. Desmond et al. [9] used coconut fiber and PLA to make microperforated plates and found that the material composition content would affect the sound absorption performance of the perforated plates, which was mainly due to the porosity and tortuous structure of the samples themselves. Dhandapania et al. [10] studied sisal fiber, palm fiber, and resin to prepare composite materials. The experiment confirmed that the composite materials had better sound absorption performance within a certain frequency range, which provided a good application for the recycling and utilization of waste hemp. Andrea et al. [11] investigated the possibility of improving the sound properties of hemp fiber composite materials through the manufacturing process. Hilal et al. [12] used waste stalks as a reinforcement for polyurethane foam composite materials and investigated the changes in mechanical properties as well as sound properties depending on the alkali treatment and reinforcement ratio of the fibers. Bioplastics and their composites based on biopolymers have broad application prospects [13]. There is a growing recognition of the need to look at the various possibilities of combining natural fibers, such as sisal, flax, hemp, and jute with polymer matrices from non-renewable waste plastics to improve the properties of fiber-based composites, as well as for potential structural and non-structural applications [14]. Fabien et al. [15] reviewed nettle and ramie fibers and their applications in biocomposites and demonstrated that nettle fibers have special stiffness and strength, which make it completely possible to prepare novel biodegradable composites.

Waste hemp stalks have a natural hollow structure and this makes them suitable for use in sound-absorbing composite materials. To broaden the application fields of waste hemp stalks, in this paper, the macromolecular, supramolecular, and morphological structures of waste hemp stalks were analyzed, and their sound absorption properties and sound absorption mechanism were discussed. Then, waste hemp stalk/polycaprolactone sound-absorbing composite materials were prepared and their sound-absorbing properties were studied. The results provide a new idea for the recycling of waste hemp stalks.

## 2. Experiment

### 2.1. Materials

The polycaprolactone powder used in this study was Perstorp 6500 (Perstorp, Malmö, Sweden). The hemp stalks came from a stalk processing plant in Shuyang, Jiangsu Province, China.

### 2.2. Equipment

An E-Model max high energy ball mill (Fuld Instruments and Equipment Co., Ltd., Suzhou, China), Model QLB-5OD/Q flat vulcanizing press machine (Rubber and Plastic Machinery Co., Ltd., Wuxi Zhongkai, Wuxi, China), JEOL JSM-6460LV scanning electron microscope (SEM; JEOL Co., Ltd., Beijing, China), and SW422/SW477 impedance tube sound absorption test system (Shengwang, Beijing, China) were used in this study.

### 2.3. Preparation of Hemp Stalk/Polycaprolactone Sound-Absorbing Composite Materials

**Figure 2:**
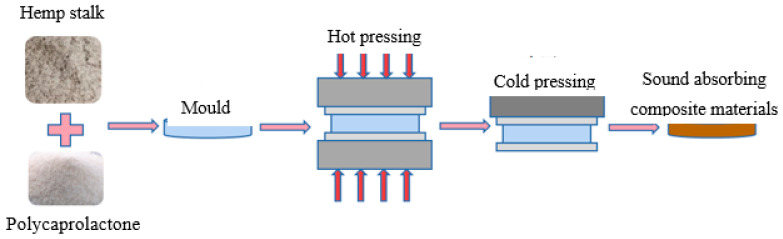
Process flow diagram for the preparation of the sound-absorbing composite materials.

The composite materials were made from a mixture of hemp stalk as reinforcement and polycaprolactone (PCL) as the matrix, measured portions of which were poured into a mold with a sprayed release agent to cast preforms 3 cm and 10 cm in diameter. The process flow diagram for the preparation of the sound-absorbing composite materials is shown in Figure 2.

The influence of four factors was investigated: hemp stalk length and mass fraction, and the density and thickness of the composite materials. The process parameters were optimized using the single-factor method. The parameters for the preparation of the sound-absorbing composite materials to be optimized are shown in Table 1.

### 2.4. Test Indicators

#### 2.4.1. Sound Absorption Coefficient

**Figure 3:**
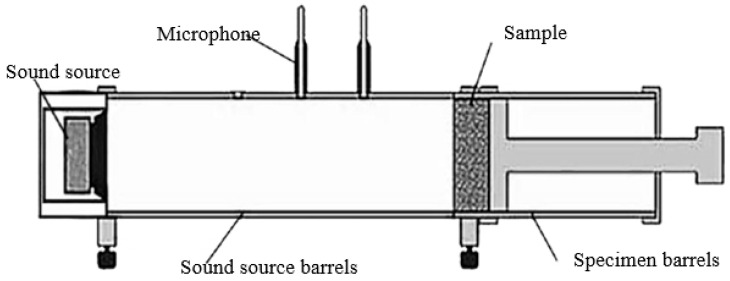
Schematic diagram of impedance tube test system.

The sound-absorbing properties of composite materials can be characterized by the sound absorption coefficient. The prepared specimens were tested at frequencies between 80–6300 Hz using an SW422/SW477 impedance tube sound-absorbing/insulation test system in accordance with GB/T 18696.2-2002 “Measurement of sound absorption coefficient and sound impedance in sound impedance tubes Part 2: Transfer function method” to obtain the sound-absorbing curve [16]. The schematic diagram of the impedance tube test system is shown in Figure 3.

The performance of the sound-absorbing materials is mainly measured by the average sound absorption coefficient (α) and noise reduction coefficient (NRC). When the value of the average sound absorption coefficient is greater than 0.2, the material qualifies as a sound-absorbing material, and when the average sound absorption coefficient is greater than 0.56, it qualifies as an efficient sound-absorbing material. The average sound absorption coefficient is the average of the absorption coefficient at six frequencies: 125, 250, 500, 1000, 2000, and 4000 Hz, and is calculated as follows:

(1) $$\alpha =\frac{\alpha_{125}+\alpha_{250}+\alpha_{500}+\alpha_{1000}+\alpha_{2000}+\alpha_{4000}}{6}$$

In addition, a material with an NRC greater than or equal to 0.2 also qualifies as a sound-absorbing material. The NRC is the average of the sound absorption coefficients at frequencies of 250, 500, 1000, and 2000 Hz, and is calculated as follows:

(2) $$\mathrm{NRC}=\frac{\alpha_{250}+\alpha_{500}+\alpha_{1000}+\alpha_{2000}}{4}$$

#### 2.4.2. Macromolecular Structure

Samples were prepared by grinding waste hemp stalks into powder using an E-Max high-energy ball mill and their macromolecular structure was tested using Fourier transform infrared spectroscopy (FTIR).

#### 2.4.3. Supramolecular Structure

An X-ray diffractometer with a Cu target, a tube current of 30 mA, a tube voltage of 40 kV, and a 2θ value range of 0–40° was used to obtain X-ray diffraction (XRD) data of the hemp stalks [17]. The peak intensity method was used to analyze the XRD data and calculate the crystallinity of the hemp stalk [18], as shown in Formula.

(3) $$X_{C}=\frac{I_{002}-I_{am}}{I_{002}}\times 100\%$$

where X_C_ is crystallinity, *I*_002_ is the maximum intensity of the lattice diffraction angle of the cellulose crystal plane, and *I_am_* is the diffraction intensity in the amorphous region.

#### 2.4.4. Morphological Structure

The morphological structure of the hemp stalks was observed in cross- and longitudinal sections under a scanning electron microscope after samples had been fixed and gold sprayed.

## 3. Results and Discussion

### 3.1. Relationships between Structural Characteristics and Sound-Absorbing Properties of Hemp Stalks

#### 3.1.1. Macromolecular Structure

**Figure 4:**
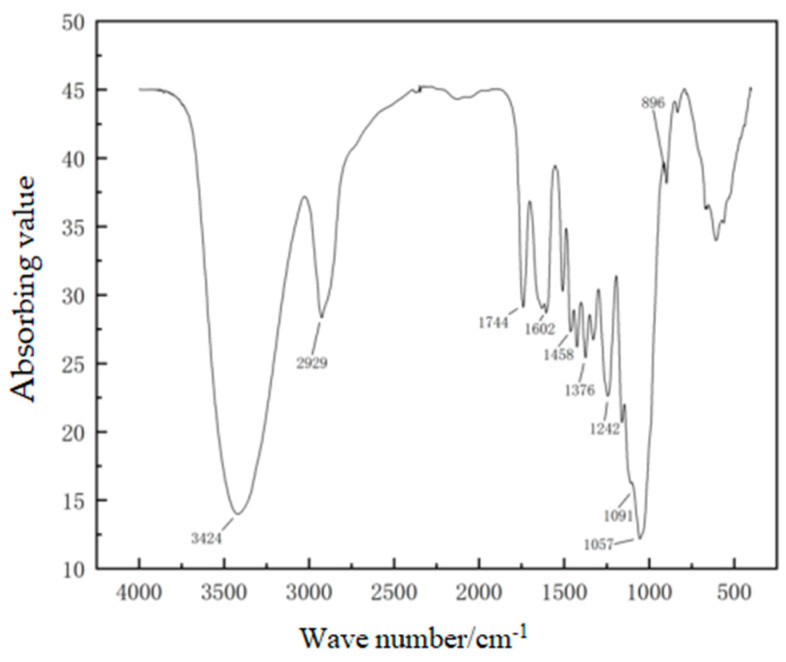
FTIR spectrum of a hemp stalk.

The macromolecular structure of the hemp stalks was investigated by FTIR, as shown in Figure 4. The attribution of the absorption bands in the infrared spectrum of the hemp stalks is given in Table 2.

Table 2 showed that hemp stalks contain cellulose, hemicellulose, and lignin, and lignin was the main component [19]. From the infrared spectrum of cellulose, there were clear characteristic absorption peaks of hydroxyl at 3424 cm^−1^, lignin carbonyl near 1744 cm^−1^, and symmetric absorbing peaks of methyl, methylene, and ethoxy at 1383 cm^−1^. The structure was dominated by -C-H and -C-O, and the absorbing peak at 1242 cm^−1^ indicated that the lignin has a lilac-based propane structure.

The above analysis shows that the hemp stalks contained a large amount of carbon and oxygen and that the macromolecular chains of hemp stalks relied on intermolecular hydrogen bonds and van der Waals forces to attract each other and maintain a relatively stable molecular structure, with a certain amount of porosity between the macromolecular chains. The pores and grooves between the molecular-chain segments increase the sound wave propagation path. When the sound waves reached the molecular chain, they caused the main chain, and functional groups such as carbonyl, hydroxyl, and methyl groups, to vibrate in response to the energy of the sound waves, which in turn causes the hydrogen bonds to move, and the segments of the macromolecular chains that could not move under the bonding of the hydrogen bonds to vibrate in response. Under the right conditions, the hydroxyl groups alternate with the main chain, and the various movements provide more friction for the propagation of sound waves; thus, they convert the sound energy into heat or other forms of energy that are consumed and, thereby, achieve a sound-absorbing effect [20].

#### 3.1.2. Supramolecular Structure

**Figure 5:**
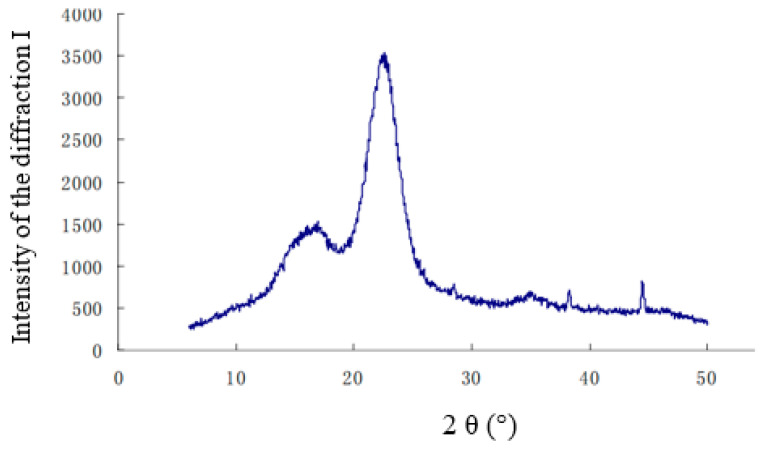
X-ray diffraction pattern of the hemp stalks.

The XRD pattern of the hemp stalks is shown in Figure 5.

In Figure 5, the main crystalline peaks in the XRD pattern are at 22–23°, and the minimum intensity is between 18–19°. The calculated crystallinity of the hemp stalks was 61.6%, which is lower than that of ramie (69.0%), flax (70.0%) [21], and other crop stalks, but similar to that of cotton stalks (59.6%). The propagation of sound waves is the result of the axial, atomic oscillations of molecules and the deformation of molecules [22]. In hemp stalk, the low degree of crystallinity and the uneven distribution of molecular weight made the molecular chains looser, and the intermolecular interaction forces are also low; thus, the mutual binding effect is weakened, and the mobility of the macromolecular chain is high, so sound waves can easily enter the macromolecular structure and be more easily consumed in the propagation process.

#### 3.1.3. Morphological Structure

**Figure 6:**
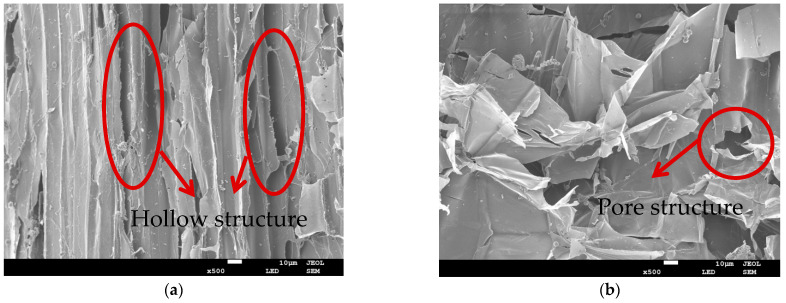

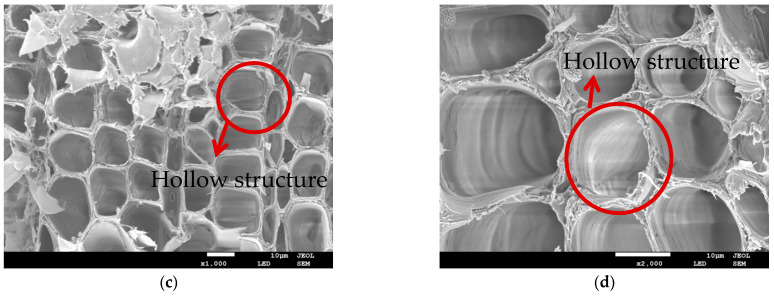
SEM of the morphological structure of hemp stalk. (**a**) Hollow structure of hemp stalk skin ×500 magnification; (**b**) Pore structure of hemp stalk core ×500 magnification; (**c**) Micromorphology of transverse section of hemp stalk ×500 magnification; (**d**) Micromorphology of transverse section of hemp stalk ×2000 magnification.

In order to investigate the relationship between the morphological structure and the sound properties of the hemp stalks, the morphological structure of the hemp stalks was investigated by SEM. In terms of physical morphology, the hemp stalk can be divided into two parts, the hemp stalk skin and the hemp stalk core, which have different structural characteristics, as shown in the SEM images of hemp stalks in Figure 6.

From Figure 6a, it can be seen that the hemp stalk skin had vertical lines, grooves, and pores. These are conducive to the absorption of sound waves. The connection between the holes improved the specific surface area of the hemp stalk, when the sound waves hit the surface of the hemp stalk, due to the large surface area, the interaction between the sound waves and the large hemp stalk is stronger and the vibration is also greater. This provides the conditions for the reflection and propagation of sound waves. As seen in Figure 6b, showing the pore structure of the hemp stalk core, the pores are highly connected and form more hollow structures. When sound waves enter this structure, due to the increased motion of air molecules in the process of propagation, the sound waves are reduced. Figure 6c,d, showing the cross-section, shows that the shape of the hemp stalk was oval and the ducts were mostly single-hole tubes. As the walls were relatively thin, they vibrate easily, and can also compress and expand. In this way, they can convert sound waves into thermal or mechanical energy.

Through the analysis of the macromolecular structure, the supramolecular structure, and the morphological structure of the hemp stalk, it can be concluded that the hemp stalk is well-suited for use in sound absorption.

### 3.2. Sound-Absorbing Composite Materials of Waste Hemp Stalks and Their Sound Absorption Properties

#### 3.2.1. Effect of Hemp Stalk Length on Sound Absorption Properties

**Figure 7:**
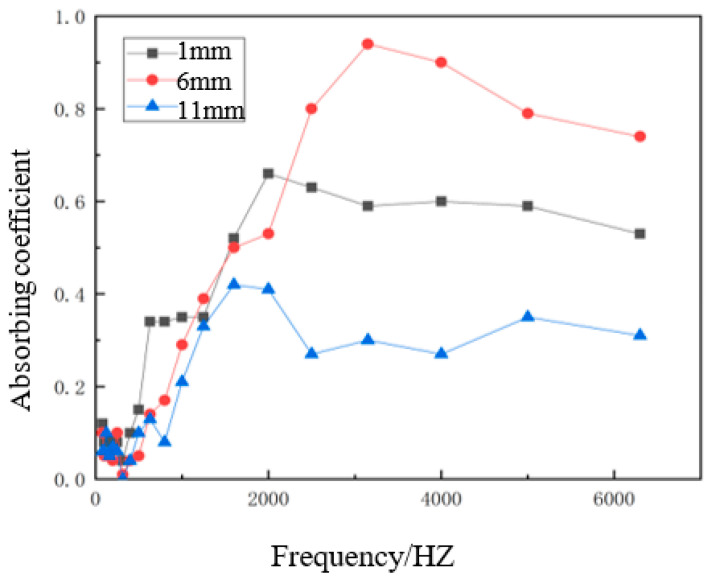
Sound absorption coefficient curves for different hemp stalk lengths.

The sound absorption coefficient curves were obtained using the impedance tube test system. The sound absorption coefficient curves of different hemp stalk lengths are shown in Figure 7.

**Figure 8:**
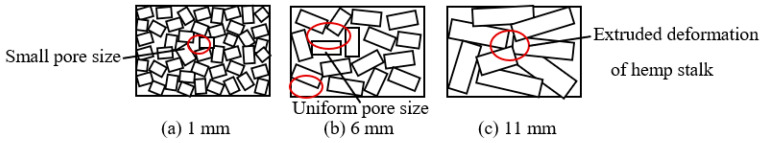
Schematic diagram of the principle behind the sound absorption performance of different stalk lengths.

From Figure 7, we can see that the overall trend of the three curves is the same, showing a decrease with the increase in stalk length. In the high-frequency region, the sound absorption performance of the composite material prepared using 11-mm lengths of hemp stalk was the worst. This is because the particles of hemp stalk are large, as were the extrusion degree of the composite material after hot pressing, and the internal cavities, leading to the reduction of effective porosity, so the consumption of sound wave in the propagation process was low. The performance of the 1-mm hemp stalk in the low-frequency region of the sound-absorbing coefficient curve was relatively good. With the 1-mm hemp stalk material, the particles were small, making for denser sound-absorbing composite materials with small pores, which resulted in increased air viscous drag. This means that, when sound waves entered the material interior, losses increased, the sound-absorbing effect was good, and the low-frequency sound wave was lost within the material, rather than through the material, Thus, the low-frequency sound-absorbing effect of the composite material prepared using 1-mm hemp stalk was better. In the high-frequency region, the sound absorption coefficient of the 6-mm hemp stalk sample was the largest, because the sound-absorbing composite material prepared using 6-mm hemp stalks was subjected to a small degree of extrusion and the pore distribution was uniform, so the sound-absorbing effect was the best. This material showed a maximum sound absorption coefficient of 0.94. Figure 8 shows the principle behind the sound absorption performance of different hemp stalk lengths.

#### 3.2.2. Influence of Hemp Stalk Mass Fraction on Sound Absorption Properties

**Figure 9:**
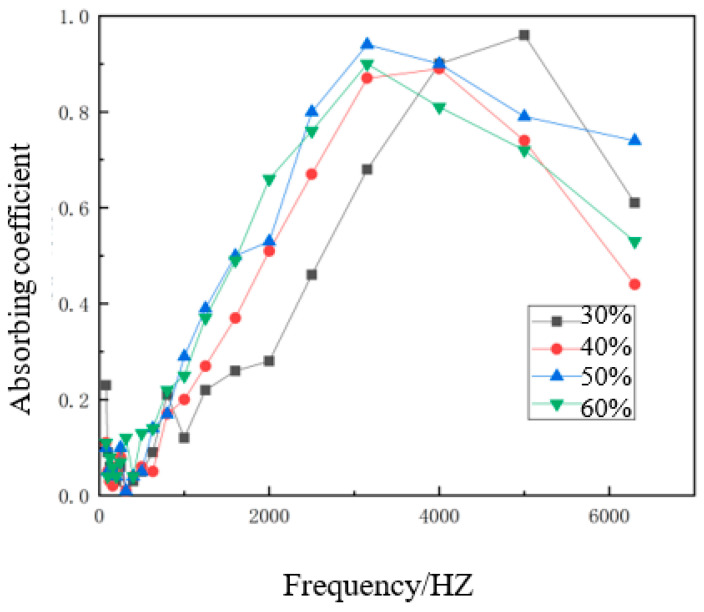
Sound absorption coefficient curves for different hemp stalk mass fractions.

With the length of the hemp stalks used in the composite set to 6 mm, the density of the composite material set to 0.30 g/cm^3^, and the thickness of the material set to 1.5 cm, the sound absorption properties of materials with hemp stalk mass fractions of 30%, 40%, 50%, and 60% were tested. The variation of sound absorption coefficient with frequency for the four sound-absorbing composite materials with different hemp stalk mass fractions is shown in Figure 9.

As shown in Figure 9, in the frequency region of 0–1000 Hz, the absorption coefficients of the four sound-absorbing composite materials were not very different, and some did not satisfy the sound absorption criterion. In the frequency region of 1000–6300 Hz, the absorption coefficient was much greater than in the low-frequency region. This was because the wavelength of the low-frequency sound wave was long and the interaction between the sound-absorbing material or structure and the low-frequency sound wave was weak, which resulted in poor sound absorption performance of the composite materials [23].

**Figure 10:**
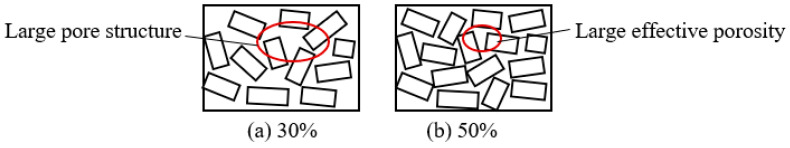
Schematic diagram of the principle behind the sound absorption performance of different hemp stalk mass fractions.

As seen from Figure 9, with the increase in hemp stalk mass fraction, almost all four curves showed a trend of first increasing and then decreasing. The reason for this phenomenon was that hemp stalks have hollow structures; the volume of hemp stalk was larger than that of polycaprolactone under the same mass, and the volume of hemp stalk was also larger. When the volume and density of the material were fixed, the hemp stalk mass fraction was low, the volume of the original composite material was small, and the hollow structure was not damaged during hot pressing so that the effective porosity of the composite material was high. When the hemp stalk mass fraction in the composite material increased, the hollow structure of the hemp stalk was destroyed by extrusion during the molding process, the interconnected pores could not form in the composite material, and the effective porosity decreased so that the sound absorption coefficient of the composite material decreased significantly. When the hemp stalk mass fraction increased slowly, the content of polycaprolactone decreased relatively, which can reduce the degree of extrusion of the hemp stalks, reduce the degree of damage to the hollow structure, and improve the sound absorption performance. The schematic diagram of the principle behind the sound absorption performance of different hemp stalk mass fractions is shown in Figure 10.

It can be seen from the figure that the sound-absorbing composite material with a 30% mass fraction of hemp stalk has a large open pore structure, low effective porosity, and poor sound-absorbing effect because sound waves can easily penetrate and pass through the material. However, the sound-absorbing composite material with a 50% hemp stalk mass fraction has a large effective porosity and absorbs more sound waves, so the sound-absorbing effect was good. Overall, the best hemp stalk mass fraction was found to be 50%.

#### 3.2.3. Effect of the Density of the Composite Materials on Sound Absorption Properties

**Figure 11:**
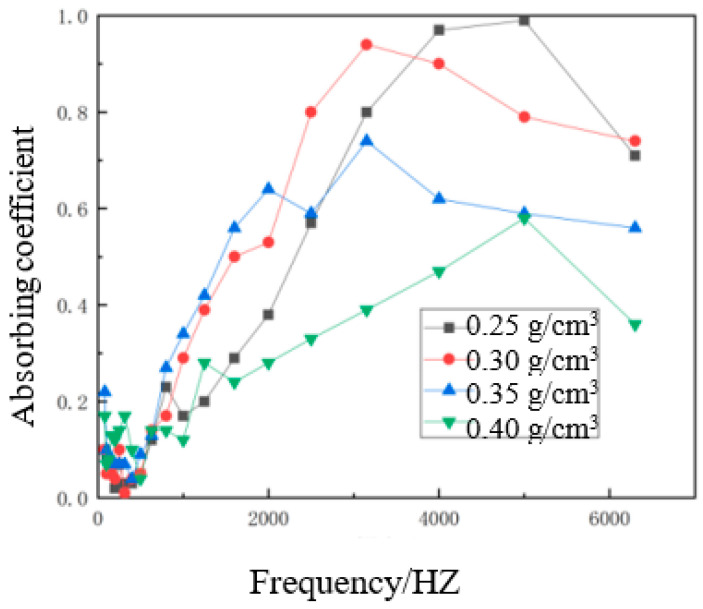
Curves of sound absorption coefficients for composite materials of different densities.

To investigate the effect of the density of the composite materials on the sound absorption properties, the sound absorption properties of waste hemp stalk/polycaprolactone sound-absorbing composite materials with different composite densities and the same hemp stalk length (6 mm), hemp stalk mass fraction (50%), and composite material thicknesses (1 cm) were tested. The variation of the sound absorption coefficient with composite material density is shown in Figure 11.

As can be seen from Figure 11, the sound absorption coefficient first increased and then decreased with the increase in the density of the composite material, and the sound absorption coefficient at high frequency was also lower. When the thickness of the material was kept constant, the effective porosity and porosity connectivity increased with the increase in the density of the material, and the sound loss in the sound-absorbing composite increased, improving its sound absorption performance. However, when the density of the material was too large, in the hot pressing process, this increased the contact area between the hemp stalks, causing closer bonding [24], leading to a decrease in the number and size of the pores, resulting in the decrease in composite material porosity, an increase in internal flow resistance and pore air drag, and sound absorption and vibration in the composite material were also reduced. The friction between the hollow structure and the density of the material leads to damage. This affects the propagation and consumption of sound waves into the interior of the sound-absorbing composite material, and, due to the full contact between hemp stalk and polycaprolactone, the number and size of the pores were constantly reduced, meaning the absorption of sound waves was also reduced, the reflection of sound waves was increased, and the sound resistance and air flow resistance of the pore openings become larger, which lead to the reduction of sound energy consumption and the deterioration of sound absorption performance. Overall, the best composite material density was 0.30 g/cm^3^.

#### 3.2.4. Effect of Material Thickness on Sound Absorption Properties

**Figure 12:**
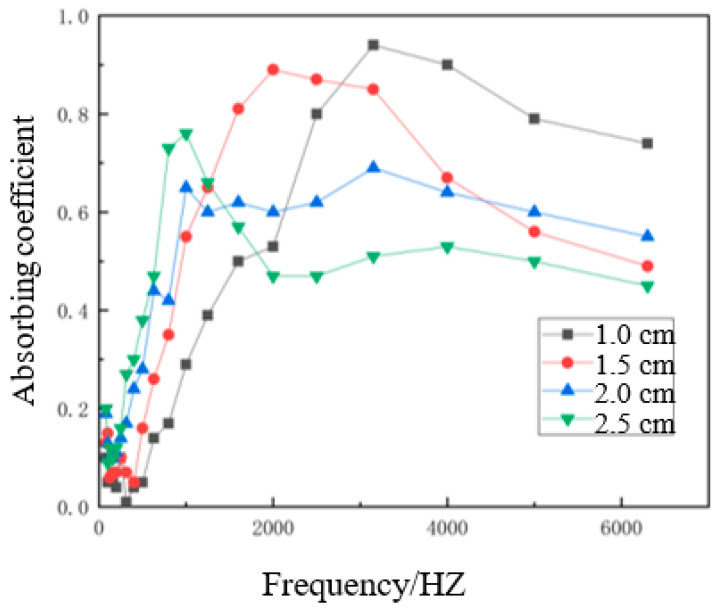
Sound absorption curves of composite materials of different thicknesses.

With the hemp stalk length fixed at 6 mm, the hemp stalk mass fraction fixed at 50%, and the density of material fixed at 0.30 g/cm^3^, the sound absorption coefficient curves of materials with different thicknesses are shown in Figure 12.

As can be seen from Figure 12, the influence of the thickness of the material on the sound-absorbing composite material was more obvious in the middle and low-frequency bands. The sound absorption performance was significantly enhanced with the increase in the thickness of material in the low-frequency band from 0–1500 Hz. Above 1500 Hz, the sound absorption coefficient curve first rises and then falls, and the high-frequency sound absorption coefficient also decreased with the increase in the thickness of the material, which was similar to the trend shown by microplates [25]. This was because, within a certain range, when the thickness of the material increases, the pore channels inside the composite material become longer, and the propagation path of the sound waves became longer. The effective porosity increased, and the long propagation path and more collisions between the sound wave and the composite material will cause more sound energy consumption; thus, the sound absorption performance of the composite material will improve. However, when the thickness of the material grows too large, the flow of air in the composite material will be affected, and the original pore channels may be blocked, reducing the effective porosity and affecting the sound absorption performance of the composite material [26]. Overall, the best performance was found with a material thickness of 1.5 cm.

#### 3.2.5. Optimal Process Samples and Comprehensive Analysis

**Figure 13:**
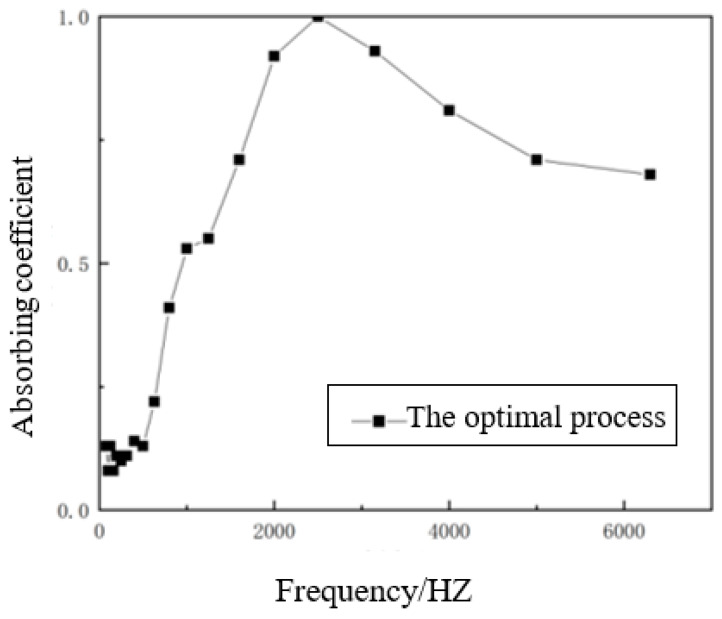
Sound absorption coefficient curve of the optimal processed composite material.

In summary, the optimal sound-absorbing process can be obtained as follows: the hemp stalk length set to 6 mm, the hemp stalk mass fraction set to 50%, the density of materials set to 0.30 g/cm^3^, the thickness of material set to 1.5 cm. Under these conditions, the average sound absorption coefficient and noise reduction coefficient were 0.44 and 0.42, respectively, and the maximum sound absorption coefficient can reach 1. The corresponding optimal processed material sound absorption coefficient curve is shown in Figure 13.

The hemp stalk length affected the pore structure inside the materials. The pores with a hemp stalk length of 6 mm were evenly distributed and had good sound absorption performance. In a certain range, with the increase in hemp stalk mass fraction, the effective porosity increased, and the sound-absorbing effect was better. The higher the density of the material, the smaller the pore structure, which was not conducive to sound propagation. Within a certain range, the greater the thickness of the material, the better the pore connectivity, the longer the propagation path of the sound wave, the higher the collision times with the material wall, and the better the sound absorption performance.

#### 3.2.6. Sound Absorption Coefficient Curves of Different Types of Reinforcers

**Figure 14:**
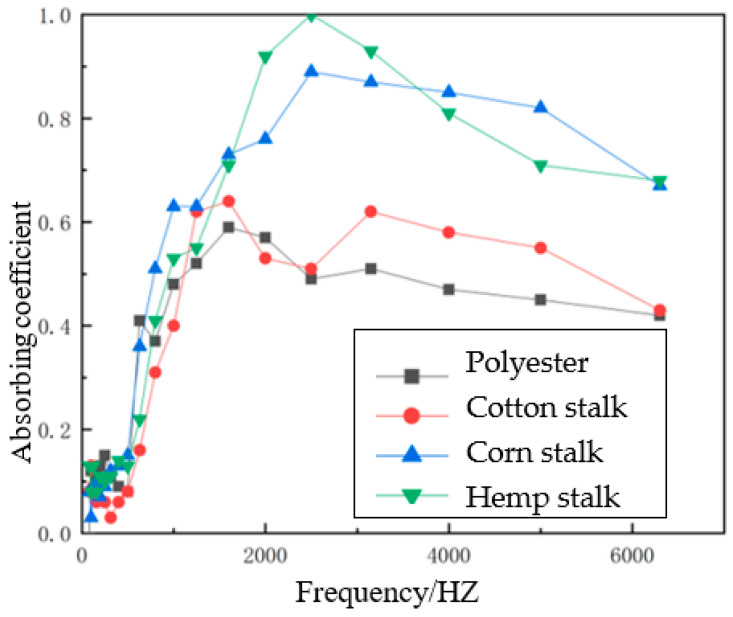
Sound absorption coefficient curve of different reinforcement materials.

Using the impedance tube test system, the sound absorption performance of different kinds of reinforcement composite materials can be tested, and the sound absorption coefficient curve obtained. Figure 14 shows the sound absorption coefficient curves of different reinforcement materials.

## 4. Conclusions

Through the analysis of the macromolecular structure and morphological structure of hemp stalks, it was found that the sound-absorbing characteristics of hemp stalks were closely related to their microstructure. From the microstructure, it can be seen that hemp stalks have a hollow tubular structure with a rough surface, around which there are many uneven pores, which are interspersed with each other. When sound waves enter the sound-absorbing composite materials, the sound waves will follow the holes into the interior of the sound-absorbing composite materials, causing gas flow and friction, thus converting part of the sound energy into heat energy, and the sound waves to be absorbed. The vibration of the sound-absorbing composite materials themselves can also absorb some of the sound waves. When the sound energy acts on a hemp stalk, the force between the chain segments, the unique hollow structure, and the large specific surface made the sound energy in the process of propagation be attenuated and converted into heat and mechanical energy, resulting in a good sound-absorbing effect.

The single-factor method was used to create sound-absorbing composite materials of waste hemp stalk and polycaprolactone, and the effects of hemp stalk length and mass fraction, and the density and thickness of the composite material, on the sound properties were investigated. With the increase in hemp stalk length, the sound absorption performance first increased and then decreased. Similarly, with the increase in hemp stalk mass fraction, the sound absorption performance first increased and then decreased. With the appropriate reduction in the density of the material, the overall sound absorption properties can be improved. The thickness of the materials had the greatest influence on their sound absorption properties, with the increase in thickness, the sound absorption properties first increased and then decreased. The final optimum process parameters of the sound-absorbing composite materials were as follows: hemp stalk length set to 6 mm, hemp stalk mass fraction set to 50%, material density set to 0.30 g/cm^3^, and material thickness set to 1.5 cm. The results showed that the average sound absorption coefficient of the optimized waste hemp stalk/polycaprolactone sound-absorbing composite material was 0.44, the noise reduction coefficient was 0.42, and the maximum sound absorption coefficient was 1.00. The sound absorption frequency band of the sound-absorbing composite materials was 100–6300 Hz, and the sound absorption mechanism followed the porous composite materials absorbing principle. 

This article confirms that waste hemp stalks have a good sound absorption performance dues to their structure and sound absorption mechanism, and provides a theoretical and experimental basis for the exploitation of waste hemp stalks to prepare high absorption coefficient, low-cost, biodegradable, environmentally friendly, pollution-free green absorbing materials, and, in doing so, help to achieve the dual goals of “carbon peak” and “carbon neutral” sustainable development. At the same time, it is recognized that, while the sound-absorbing composite materials prepared in this paper can be applied to places that need noise reduction, their low-frequency sound-absorbing effect is not very good, so they are not suitable for use in all types of sites.

## Figures and Tables

**Table 1:** The parameters for the preparation of the sound-absorbing composite materials to be optimized.

Factors				
	Hemp Stalk Length	Hemp Stalk Mass Fraction	Density of Materials	Thickness of Materials
Level	1 mm	30%	0.25 g/cm^3^	1.0 cm
	6 mm	40%	0.30 g/cm^3^	1.5 cm
	11 mm	50%	0.35 g/cm^3^	2.0 cm
		60%	0.40 g/cm^3^	2.5 cm

**Table 2:** Attribution of absorption bands in the FTIR spectrum of a hemp stalk.

Wave Number/cm^−1^	Absorbing Band Attribution and Description
3424	O-H stretching vibration
2929	C-H stretching vibrations (polysaccharides, lignin, fatty acids, saturated hydroxyl groups)
1744	C=O stretching vibrations (carboxylic acids, esters, anhydrides, aldehydes, ketones, amides, acyl halides, etc.)
1602	Carbon skeleton vibrations of benzene rings (lignin)
1458	C-H bending vibrations (lignin, CH in polysaccharides) and benzene ring skeleton vibrations (lignin)
1376	C-H In-plane bending vibration (cellulose and hemicellulose)
1242	Guaiacyl propane structure and acyl-oxygen bond CO-O stretching vibrations
1091	C-O stretch, secondary alcohols, and aliphatic ethers
1057	Alkoxy bond stretching vibrations and C-O stretching vibrations in acetyl groups (cellulose and hemicellulose)

## Data Availability

The data presented in this study are available on request from the corresponding author.
